# Online behaviour change technique training to support healthcare staff ‘Make Every Contact Count’

**DOI:** 10.1186/s12913-020-05264-9

**Published:** 2020-05-07

**Authors:** Anna Chisholm, Lucie Byrne-Davis, Sarah Peters, Jane Beenstock, Suzanne Gilman, Jo Hart

**Affiliations:** 1 Department of Psychological Sciences, University of Liverpool, Liverpool, L69 7ZA UK; 2 Division of Medical Education, University of Manchester, Manchester, M13 9PT UK; 3 Division of Psychology & Mental Health, University of Manchester, Manchester, M13 9PL UK; 4 Lancashire Care NHS Foundation Trust, Preston, PR5 6AW UK; 5 Health Research, Lancaster University, Lancaster, LA1 4YW UK; 6 Division of Medical Education, University of Manchester, Manchester, M13 9PT UK

**Keywords:** Behaviour change, Staff training, Communication skills

## Abstract

**Background:**

National Health Service (NHS) staff support service users to change health-related behaviours such as smoking, alcohol consumption, diet and physical activity. It can be challenging to discuss behaviour changes with service users hence training is needed to equip staff with up-to-date, evidence-based behaviour change skills. In order to identify how training may help to improve health professional skills in this area, this study evaluated change in professionals’ behavioural determinants following an online behaviour change skills module as part of Making Every Contact Count (MECC) training.

**Methods:**

This evaluation comprised a within-subject design in which staff from one Northwest England NHS Trust completed a 9-item survey immediately before and after training. This prospective survey identified behavioural determinants regarding adhering to MECC recommendations to hold health conversations with service users and provided written comments about their training experiences. Individuals working within the Trust in clinical or non-clinical roles were eligible to take part and were invited to contribute to the evaluation upon uptake of their usual NHS staff online training programmes.

**Results:**

Of participants completing the evaluation (n=206), 12 professional cadres accessed the module, most being female (91%), nurses/midwives (43%), working in children and family services (48%), aged 22 - 62 years. Eight behavioural determinants increased significantly following training, with effect sizes ranging from sizes ranging from 0.27 to 0.51; ‘identity’ did not change. Content analysis of written feedback (n=256) indicates that training enhanced staff behaviour change skills, modelled a productive and specific method of adopting a patient-led approach to behaviour change conversations, and identified that staff may require further support with embedding skills in practice.

**Conclusions:**

Behaviour change science can be translated into useful learning for NHS staff. Online training can engage staff in learning about behaviour change skills and increase their behavioural determinants to adopt these skills in practice.

## Background

Health care professionals face an on-going challenge to meet the demands of a growing and aging population whilst increasing their focus on illness prevention and health promotion [1, 2]. It is widely acknowledged that making changes to key health-related behaviours including smoking, alcohol, food consumption and physical activity, could have a substantial impact on individual and population health outcomes, and on health care costs [1, 3–5]. All national health service provider organisations in England are contractually obliged to train their staff to have opportunistic conversations with patients and carers making health behaviour changes that could impact positively on their health. The approach called ‘Making Every Contact Count’ (MECC) has not included a consistent approach to training and there is a paucity of evidence to support the effectiveness of MECC, both in terms of professional behaviour being changed by training and patient behaviour being changed by more healthy conversations [6, 7]. Unsurprisingly, MECC leads also report variations in implementation success [8].

Evidence illustrates that health professionals often encounter challenges to engaging in discussions with people about making health-related behavioural changes. Common barriers include feeling unskilled in this area, lacking confidence to address behaviour change, doubting the utility of such conversations, or fearing that it would cause offence or damage relationships with individuals, leading to disengagement with services [9–12]. Further, it is demonstrated that those responsible for designing and delivering health behaviour education to health professionals can also feel unclear about what to include, with some highlighting difficulty teaching trainees about behaviour change because they struggle with this themselves in practice [13].

To overcome these challenges, and provide clarity on best practice in designing and delivering behaviour change training for National Health Service (NHS) staff, training must be informed by current evidence and understanding about what strategies are most likely to be effective *and* implementable within current NHS settings. Furthering this line of understanding requires conducting evaluations that can demonstrate the impact training is likely to have on staff behaviour, including their ability to learn behaviour change skills and put them into practice. Although there is evidence for the effectiveness of using brief interventions within healthcare consultations to support service users to stop smoking,[14] and reduce weight, [15] it is less clear how best to assist the wider NHS workforce with adopting and fully implementing behaviour change approaches across a range of settings and addressing a range of behaviours, as advocated by MECC.

As with patient-facing behaviour change interventions, it is important to identify mechanisms of action accounting for how training might work to influence health professionals’ clinical practice by assessing change in behavioural determinants; including their Capability, Opportunity, and Motivation towards a given Behaviour (COM-B framework) [16]. A range of determinants from various behavioural and social cognition models identify key theoretical constructs that fit within the broader COM-B system such as self-efficacy, subjective norms, behavioural attitudes, outcome expectancies, action plan, action control, identity, and behavioural expectations [17, 18]. Research demonstrates that COM-B framework factors can explain health professional behaviours in a variety of settings such as implementing children’s health screening, and providing contraception advice [19, 20] and this efficacy in explanation of health professional behaviours makes it an ideal framework for understanding change in determinants following a training programme.

With regards to MECC training, no research has investigated how it might influence staff behavioural determinants. However, one evaluation of an existing United Kingdom (UK) based MECC training intervention to equip health professionals with ‘Healthy Conversations Skills’, [21] shows promise in terms of its ability to improve short, medium and long-term outcomes. However, without investigating behavioural determinants alongside behavioural outcomes, interventions cannot be fully replicated or compared, and understanding why or how training may work to change NHS healthcare practice remains unclear. This study therefore aimed to investigate behavioural factors related to NHS staff’s capability, opportunity, and motivation to engage in health conversations with service users in healthcare settings, following completion of an online behaviour change training module.

## Methods

### Design

Given that training was delivered online, it was deemed most appropriate to adopt a prospective survey design in order to minimise possible recall bias and maximise staff uptake at the time training was conducted. Prompting data collection as close as possible to training completion increased the likely validity of measures taken by reducing possible recall bias or influence of uncontrolled confounding factors (e.g. additional training / discussion with colleagues). To enable as complete an understanding as possible about the likely influence of the training, the evaluation included both qualitative and quantitative components, to explore the self-reported training experiences of staff and change in their behavioural determinants, following the online module.

### Participants and procedure

NHS staff, including clinical and non-clinical employees, working in a large Northwest England NHS mental health and community trust, were invited via their network directors and a trust-wide email news briefing to take part in a 40-minute online MECC behaviour change training module. Evaluation measures were embedded within the module as part of an existing service evaluation and thus participants were automatically directed to the pre- and post-training assessments though completion of them was voluntary. Ethical approval was not required due to this data being routinely collected as part of an on-going service evaluation [22].

The online module content and evaluation measures were directly informed by an existing training intervention based on theoretically-informed behaviour change techniques (see http://www.tentpegs.info) [23, 24] and was made available to staff by the NHS trust as part of regular training opportunity announcements. Module sections included: (i) ‘Introduction’ (describing the wider context of the training, how its learning objectives fit with MECC and highlighting its relevance to trainees), (ii) ‘Reducing Resistance‘ (introducing clinical communication skills including eliciting individuals' Ideas, Concerns and Expectations [ICE], using reflection and empathy), (iii) ‘Understanding Behaviour’ (complexity of health behaviour, COM-B, and impulsivity), (iv) ‘TEnT PEGS toolkit’ (behaviour change conversation pitfalls including scare tactics and information overloading; behaviour change techniques which can be used in conversations – TEnT PEGS toolkit; (v) ‘If-Then Plans’ (implementation intentions and action planning). Each section included written information and interactive activities including multiple choice and open answer questions based on various patient scenarios. In line with pedagogical evidence, we designed interactive activities which (a) highlighted the relevance of the educational content to the participant, (b) gave participants the opportunity to apply learned knowledge and skills to enhance mastery and self-efficacy, and (c) provided tests of acquired knowledge to enhance learning [25]. In addition to this, two researchers (AC & JL) independently coded the module learning content for behaviour change techniques (BCTs) as defined by a current BCT taxonomy [26]. Coders obtained ‘almost perfect’ inter-rater reliability [27] (Kappa = 0.87) and identified 11 BCTs in total within the course content (Table 1).

**Table 1:** Behaviour change techniques included within online training for NHS staff

Behaviour change technique	Technique description within training content	Illustrative example
Goals setting (outcome)	Expected learner outcomes from the course are specified at outset of the course	*“The worker is able [following training] to select and use brief lifestyle behaviour change techniques that help individuals take action about their lifestyle behaviour choices … ”*
Feedback on behaviour	Interactive activities involve the learner selecting BCTs for different patient cases; feedback on the appropriateness of their selection is provided for each case.	*“That’s correct. You’ve chosen the best technique for this service user. You’ve shown that you can listen for cues and adopt the most helpful technique to bring about behaviour change”*
Instruction on how to perform the behaviour	Information is provided explain to learners how tailor behaviour change interventions to service users	*“You can ensure that each intervention that you create is specific to the individual by following a three-step process during behaviour change discussions: 1. Listen out for cues from the service user. 2. Select the TEnT PEGS category based upon the identified cue. 3. Use the behaviour change technique suggested within the selected category … ”*
Information about antecedents	Explanations about how good communication skills can lead to more constructive behaviour change conversations with service users and how poor communication skills can prevent this.	*“Difficult conversations are made easier by excellent communication. Reducing resistance before you start a sensitive topic can make service users more receptive to thinking about behaviour change.”*
Information about consequences	Description of the positive consequences of using TEnT PEGS BCTs in practice including taking into account patient needs, recognising cues, tailoring the behaviour change intervention, and working in a patient-centred way.	*“Although the TEnT PEGS toolkit focuses on techniques to use during behaviour change discussions, it also offers strategies for initiating behaviour change conversations in a collaborative manner, and can help you implement on-the-spot behaviour change techniques opportunistically within standard consultations”*
Demonstration of behaviour	Learners are shown written if-then plans that match presented patient cases in order to show them how they might use this technique with a service user.	*“You might have written something like … If Ken is eating meat, then he will take the skin off / If Ken is doing his weekly shop, then he will make sure he buys at least five different kinds of fruit and vegetables … ”*
Behavioural practice/ rehearsal	During interactive activities, the learner choses a specific response to a presented patient case, in order to practice selecting BCTs based on patient cues.	*“How would you help Ken? Which of the following strategies would you suggest to help Ken lose some weight?” [7 strategies are presented; learners select all that apply]*
Social reward	Learners are congratulated for successfully completing the course and learning new skills	*“Well done! You’ve now completed this module on TEnT PEGS. You should feel more familiar with: how to reduce resistance to change; the techniques detailed in the TEnT PEGS toolkit; how to formulate if-then plans … ”*
Verbal persuasion about capability	During the course and upon completion, learners are reminded that they will be able to put learned skills into practice with service users.	*“Each method will help you to think about structuring a conversation and will help you to have more productive conversations with service users”*
Focus on past success	Learners are reminded at the outset of the course that they have previously obtained relevant skills related to this topic	*“Some of you already have prior knowledge and skills about behaviour change. This e-learning programme will refresh your learning and provide a practical resource of behaviour change techniques … .”*

### Measures

#### Survey

An electronic survey was administered to participants immediately before and immediately after completing the training module, to assess a range of theoretically driven factors that relate to staff likeliness to go on to hold health behaviour change conversations with service users in practice. Items were selected by the authors following a narrative review of current evidence about key predictors of health professional behavioural change. Each item was chosen in line with theories and models of behaviour that have been shown to have predictive value with regards to *clinicians’* intentions and behaviours [28]. Prior to training 10-items were used to assess: (i) an estimation of baseline behaviour (number of health conversations with service users in the preceding week); (ii) staffs’ behavioural determinants including self-efficacy, subjective norms, opportunity, behavioural attitudes, outcome expectancies, action plan, action control, and self-identity (assessed via 7-point Likert scales, strongly agree – strongly disagree); examples of items are ‘It is part of my role to have meaningful conversations with service users to help them make necessary lifestyle changes’ (identity) ‘I am confident in my ability to have meaningful conversations with service users to help them make necessary lifestyle changes’ (self-efficacy) and (iii) staff’s behavioural expectations (of every 10 service users, how many would staff expect to have lifestyle change conversations with (for full survey see Supplementary File 1). Since the evaluation measure needed to be concise the accepted standard of providing four or five items per construct could not be met [29]. Therefore, each behavioural determinant was assessed using only one question and reliability statistics are therefore not available. To maximise content validity, we constructed items using accepted techniques described in the REBEQI manual and that have been used previously to measure these determinants [30]. Following training, all items except the baseline estimation of behaviour were repeated.

#### Free text feedback

In order to capture unanticipated learning outcomes and perceptions of the module participants were asked upon completion, to provide qualitative data in the form of open-text written feedback about their experiences and views of the session by responding to the following three questions: *What do you feel you’ve learned in this session? What might you use or not use in practice? Do you have any other comments about this session?*

### Analysis

Survey: Descriptive statistics were used to describe the study sample. This derived from available demographic data relating to staff age, sex, cadre, and clinical network affiliation. Wilcoxon Signed Rank Tests were conducted following normality tests, to assess within-group differences in staff’s behavioural determinant ratings pre- and post-training and effect sizes were calculated for each pre-post comparison. Missing data was minimal (<5% on 15/18 pre- and post-training variables); however multiple imputation was performed on three variables with >5% missing data using IBM SPSS software package.

Free text feedback: Qualitative data obtained via written feedback from module completers was analysed using conventional content analysis [31] whereby one author (AC) coded all responses using inductive category labels. Two other authors (SP & JH) then independently coded 20% of responses in order to examine validity of category labels and coding. Coding was refined through discussion following this exercise and organised hierarchically into broad themes, categories and sub-categories that were not pre-determined by the research team and that were deemed to best represent identified patterns in the data.

## Results

### Participants

Of 482 staff who took the module, 206 (43%) completed pre- and post-training surveys. Twelve different clinical and non-clinical professional cadres accessed the online module. Most participants were female (91%), nurses or midwives (43%), working in children and family service settings (48%) and were aged between 22 and 62 years. On average staff reported holding eight behaviour change conversations per week with services users (Range = 0 - 85) (assessed at baseline). Table 2 describes full sample characteristics.

**Table 2:** Demographics for participants completing pre- and post-training surveys (n=206)

Participant characteristic		Descriptive statistics (n=206)
		*Mean (SD), Range*
Age (years)		44 (9.4), 22-62
Self-reported behaviour change conversations held per week		8 (10.6), 0 – 85
		*N (%)*
Cadre	Nursing & midwifery registered	89 (43%)
	Medical & dental	40 (19%)
	Allied health professional	22 (11%)
	Specialist practitioner	16 (8%)
	Additional clinical services	12 (6%)
	Administrative & clerical	9 (4%)
	Practitioner	7 (3%)
	Psychological therapist (qualified)	2 (1%)
	Technician	2 (1%)
	Social worker	1 (<1%)
	Advanced practitioner	1 (<1%)
	Healthcare Scientist	1 (<1%)
	Missing	4 (2%)
Clinical network	Children and families’ services	99 (48%)
	Adult community services	80 (39%)
	Adult mental health	18 (9%)
	Secure services	5 (2%)
	Corporate services	1 (<1%)
	Missing	3 (2%)
Gender	Female	187 (91%)
	Male	13 (6%)
	Prefer not to say	4 (3%)

### Behavioural determinants for having health conversations with service users

Within-group comparisons indicated that all variables were found to be higher post-training except for identity, which was the same pre- to post-training. Pre training means were relatively high (means 5.2-5.9 on a 1-7 scale where 1 is strongly disagree to 7 strongly agree) for ‘self-efficacy’, ‘subjective norm’, ‘behavioural attitudes’, ‘outcome expectancies’, ‘action control’ and ‘identity’. Significant increases were observed for all these factors post training apart from ‘identity’ (see Table 3). ‘Action planning’ and ‘perceived behavioural control’ were relatively lower (means 4.4 and 4.5) but both increased significantly post training. Effect sizes (r) ranged from 0.27 to 0.51 (mean = 0.37). Observed effect sizes were *low* for ‘self-efficacy’ and ‘subjective norm’, *medium* for ‘perceived behavioural control’, ‘behavioural attitudes’, ‘action control’ and ‘outcome expectancies’, and *high* for ‘action plan’ and ‘behavioural expectation’. Table 3 displays full within-group comparisons.

**Table 3:** Within-group comparisons for participants completing pre- and post-training surveys (n=206)

Behavioural Determinant		Pre-training		Post-training		Effect size (r)
	Scale	Mean (SD)	Median (IQR)	Mean (SD)	Median (IQR)	
Self-efficacy*	1-7	5.6 (1.2)	6.0 (1)	6.0 (1.0)	6.0 (1)	0.27 ^**S**^
Subjective norm*	1-7	5.5 (1.3)	6.0 (2)	5.9 (1.1)	6.0 (1)	0.29 ^**S**^
Perceived behavioural control*	1-7	4.5 (1.5)	5.0 (3)	5.0 (1.6)	6.0 (2)	0.37^**M**^
Behavioural attitudes*	1-7	5.2 (1.2)	6.0 (2)	5.6 (1.1)	6.0 (1)	0.39 ^**M**^
Outcome expectancies*	1-7	5.3 (1.1)	6.0 (1)	5.8 (1.0)	6.0 (2)	0.51 ^**L**^
Action plan*	1-7	4.4 (1.6)	5.0 (3)	5.2 (1.4)	6.0 (2)	0.50 ^**L**^
Action control*	1-7	5.3 (1.1)	6.0 (1)	5.8 (1.0)	6.0 (0)	0.33 ^**M**^
Identity	1-7	5.9 (1.1)	6.0 (1)	6.0 (1.1)	6.0 (1)	N/A
Behavioural expectation*	1-10	5.9 (3.0)	6.0 (5)	6.6 (2.9)	7.0 (5)	0.33 ^**M**^

*p<.001; ^S^ = Small effect size; ^M^ = Medium effect size; ^L^ = Large effect size.

### Behavioural expectations to engage in health conversations

For 206 participants, the average behavioural expectation ratings - i.e. out of ten clients they saw, how many they expected to have health conversations with were, Mean=6.26 (S.D. = 3.0), Median=7 (Interquartile range = 0-10) and Mode=10. These figures indicate that there are a group of people (24%) who expect to have healthy conversations with all service users, with the other 76% of the sample normally distributed around a mean and median of approximately five, indicating expectations to engage in health conversations with around half of the service users they see. Given this, in addition to our planned analyses, we conducted a *post-hoc* analysis to identify change in participant scores from pre- to post-training when excluding those who expected to have conversations all the time 10/10 service users and who were therefore not amenable to any increase in behavioural expectation. However, these analyses indicated no changes to the results presented above with the full sample.

### Content analysis of staff views and experiences of the online module

All staff completing the module were provided the opportunity to provide written feedback, regardless of completing the pre- or post-training surveys. Thus, a total of 256 of 482 staff who completed the module submitted feedback on their experiences and views of the session. Content analysis identified three key themes accounting for these data: (1) *Learning from the session*, (2) *Impact of the session for the individual*, (3) *Views on session components*. Each theme is described below in terms of the categories and sub-categories that reside within each theme. Table 4 displays the full thematic structure alongside illustrative quotes from the data evidencing each sub-category.

1. Learning from the session

**Table 4:** Content analysis structure and illustrative quotes

Theme	Category	Sub-categories	Sub-category illustrative quotes (participant ID number)
**(1) Learning from the session**	Behaviour change techniques (n=106)	*Patient-led (n=20)* *If-then plans (n=16)* *Tent Pegs (n=15)* *Goal setting (n=5)* *Avoid dictating (n=5)* *Positive reinforcement (n=3)* *Address barriers to change (n=2)* *Reduce resistance (n=1)* *Feedback (n=1)* *Habit formation (n=1)*	*To be listening for the cues from the individual (22)* *Will definitely use the If and then way of planning (203)* *Very useful techniques; especially helpful TEnT PEGS (83)* *More positive in setting goals (43)* *I will not tell service users what to do instead I will work on their ideas (220)* *Continuing to use positive reinforcement (193)* *To explore barriers more (136)* *How to reduce resistance (83)* *Provide feedback (88)* *How simple changes can make a big difference in changing habits (234)*
	Communication skills (n=49)	*Raising the issue (n=14)* *Questioning style (n=6)* *Listening (n=5)* *Non-confrontational (n=2)* *Building rapport (n=1)*	*Great techniques how to start conversations (241)* *How to phrase questions in a better way (162)* *I have learnt how to listen more carefully at what is being said (140)* *Get them (service users) to think about their lives in a nice gentle way (75)* *To engage with service users (35)*
	New knowledge (n=20)	*Best behaviour change methods (n=7)* *Broadened skills (n=4)* *Theory behind practice (n=3)* *Meet MECC recommendations (n-1)* *Professional development (n=1)*	*Understand more the best way to help service users to change (14)* *It has broadened my approach in helping service users (103)* *Very interesting to understand the theory behind the practices we use (30)* *I now have the information to help me with MECC (9)* *Knowledge and information for my professional development (186)*
	Refreshed previous knowledge/ skills (n=16)	*Motivational Interviewing (n=6)* *Reinforces current practice (n=4)* *Prior lifestyle change training (n=2)*	*Reinforced motivational interviewing techniques (194)* *All of it to enhance what I already deliver (164)* *Reinforced learning I have already had re behaviour change (183)*
	No new learning (n=8)	*Already do this (n=6)*	*The session was good however I use most of the suggested techniques naturally (110)*
**(2) Impact of session for the individual**	Intention to implement in practice (n=27)	*Help patients change (n=9)* *Help colleagues change (n=5)* *Help self change (n=3)*	*I will definitely use these interventions in my practice (118)* *Can be applied to my day to day conversations with colleagues and service users (253)* *I have learnt to take more control over my unhealthy habits (18)*
	Reflection on practice (n=22)	*Value of change discussions (n=12)* *Adapt for my area of work (n=3)* *Broadened views on role (n=2)* *Recognise use of effective techniques (n=1)* *Drawn attention to topic (n=1)*	*The importance of having conversations about lifestyle change (67)* *These may require adaptation for the service users I work with to enable them to understand and participate in the process (78)* *I will continue to always address healthy lifestyles and behaviour change with everyone, not just my clients (213)* *What techniques I am already using that are considered good in prompting behaviour change (200)* *It has just made me think more about supporting clients through behaviour change (159)*
	Confidence (n=19)	*Discussing lifestyle topics (n=14)* *Makes current practice easier (n=2)* *Undermined confidence (n=1)*	*Feel much more confident in making the lifestyle changes conversations with patients (83)* *I have learned easier ways of how to broach the subject of lifestyle changes (209)* *It actually undermined confidence as module very regimented (101)*
**(3) Views on session components**	Generally valuable (n=28)	*Useful (n=6)* *Informative (n=6)* *Interesting (n=4)* *Insightful (n=3)* *Awareness (n=2)*	*Very useful (217)* *I found this session extremely informative (165)* *I have found session very interesting (15)* *Feel it has given me more insight (149)* *More awareness (150)*
	Content (n=26)	*Relevant to practice (n=7)* *Not relevant to current role (n=7)* *Confusing / Complex (n=6)* *More support to implement (n=2)* *Enjoyed theory (n=2)* *Partially relevant to role (n=1)*	*All information very useful for daily practice (2)* *Wouldn't use in my role but very thought provoking (154)* *Lot of information confusing (166)* *Good but I will need more working practice of it (158)* *I enjoyed the theory side of this course (249)* *It can be used to help with specific changes to behaviours covered by our specialty but the inference is now that we are supposed to tackle all aspects of healthy living and that is way beyond our scope (102)*
	Delivery style (n=10)	*Prefer face-to-face (n=3)* *Technical difficulties (n=2)* *External distractions (n=1)* *Interaction useful (n=1)*	*Not the easiest mode of education for myself, prefer hands on learning (182)* *Difficulties with the programme running slow (221)* *Found it quite difficult to concentrate in the office environment (218)* *Good use of interaction (29)*
	Structure (n=12)	*Difficult to navigate (n=9)* *Too lengthy (n=6)*	*Quite confusing and difficult to navigate. Not user friendly. (237)* *Very long and time consuming (48)*

Frequency of sub-category responses do not necessarily add up to totals of category responses as broader quotes could be coded at category level only, whereas sub-category titles reflect those that went on to provide more specific detail on category level responses. MECC = Making Every Contact Count. Not able to code 24 responses

Staff identified learning multiple behaviour change techniques (BCTs) from the online module such as if-then plans, goal setting, feedback, and habit formation. In line with the TEnT PEGS central philosophy, staff most commonly emphasised learning the value of (and how to) adopt a patient-led approach during behaviour change conversations – i.e. recognising what technique to use when. Staff also highlighted learning specific communication skills such as initiating change conversations, phrasing questions appropriately and listening to service users. As well as identifying areas of new knowledge gained, staff also felt the module helped them to refresh and consolidate previous knowledge acquired through other training. A minority of staff identified not learning anything new from the course perceiving that they already adequately conduct behaviour change conversations in practice.

(2) Impact of the session for the individual

Staff highlighted their intention to change via implementing their learning from the module into their day-to-day practice, and specified that they would use this to help service users make changes to aspects of their lifestyles, but also that they would use these skills to help colleagues with behaviour change, and themselves. Staff found the module led them to reflect on their practice and in particular emphasised how it made them think about the value of behaviour change conversations with service users, what their role entails with regards to this, how the module content could be adapted for their particular area of work, and what techniques they are already using which would be considered good practice in relation to this module. Finally, staff indicated that this training enhanced their confidence to discuss lifestyle topics with service users, making these conversations easier for some, although one individual felt that the module undermined their confidence as the content was too rigid in its approach.

(3) Views on session components

Many staff commented that the session was valuable to them in terms of being useful, informative and interesting. Although many staff found the module content relevant to their practice, others felt it did not relate to their day-to-day practice and so were less likely to implement their learning with service users. Some found their learning was impeded by the content being confusing or complex in places, and some noted that the structure was difficult to navigate and too lengthy. A minority felt they needed further opportunities to be able to translate their learning to practice and commented that they would have preferred face-to-face training or were impeded by external distractions or technical difficulties due to the online format.

## Discussion

### Main findings of this study

The study findings show that after completing a 40-minute online behaviour change training module, a positive increase was observed in a range of theoretical constructs that determine the likelihood that NHS staff will adhere to MECC recommendations and engage in health conversations with service users. The exception was that staff beliefs that healthy conversations are part of their role (‘identity’) was high prior to training, and remained high following it. This indicates that uptake for this training includes staff who already feel this should be a part of their role. However, there was scope in this self-selected group for improvement on all other theoretically driven behavioural determinants measured (self-efficacy, subjective norms, opportunity, attitudes, outcome expectancies, action plan, action control), and behavioural expectations. The qualitative findings also indicate that the module provides many staff with a broad range of new and reinforced behaviour change and communication skills, and impacts upon their practice and confidence to practice in this area. However, these data show that even in this potentially highly motivated group, there are still a number of potential barriers to staff being able to translate this learning to practice that should be taken into account including: overlap with previous training or skills; perceived relevance to current role; and limitations of the online format. Additionally, in keeping with previous studies that show that staff do not routinely have healthy conversations, the mean number of service users with whom the majority of this group expected, pre-course, to have healthy conversations, was only 50%.

### What is already known on this topic and what this study adds

All NHS staff members are expected to implement MECC in their practice but many can find this challenging and a range of barriers exist preventing organisations from fully embedding such recommendations within health care practice [8]. Research shows the types of theoretical constructs that are known to determine staff practice behaviours, [16, 19, 30] and it can be argued that this training could enhance the implementation of MECC recommendations in practice via increasing these factors that would support staff in having health conversations with service users. Although further research observing staff behaviours in practice settings is required to support this, the current findings provide evidence about *how* this change in staff behaviour does occur, if indeed it does occur in real world settings. This is particularly valuable as this builds upon previous research which suggests that similar training could improve skills but doesn’t identify potential mechanisms of change [21].

### Limitations of this study

A limitation of this work which is common within medical education research, [32] is that it was not possible to include a control group within the study design and thus we cannot draw firm conclusions that the observed improvements in staff’s behavioural determinants and expectations are due to training completion. The possibility exists that change in these constructs may have occurred due to other confounding factors, for example prompting reflection on staff practice, or being exposed to general information about preventive health care. However, this study does provide a good indication of the expected amount of outcome variance that could be observed in a larger more controlled evaluation study. Thus this study could inform a future large-scale evaluation of this training. The results also indicate possible ceiling effects with high average pre-test scores, and as raw score changes, though significant were relatively small, this provides an indication that broader scales and/or more specific questioning in future work may be even more helpful in identifying the extent of change and type of change occurring in staff skill sets. Finally, data were not obtained to identify exact post-training data collection times which risks that if recall bias existed it was undetected. Future research should also explore how staff use and experience the online module itself. This would enable further understanding about how useful, relevant, usable and engaging the included training content is (thus further identifying potential active/inactive components of the intervention). This would also indicate how module uptake could be enhanced and achieve broader reach, particularly to staff who are less likely to identify health conversations as part of their role.

## Conclusion

This study identifies that theory-informed online training could improve staff engagement (including motivation, perceived capability and opportunity) in health conversations with service users. Training of this kind should be further evaluated within a more controlled study design to contribute to the evidence base for MECC and crucially improving understanding regarding how best to equip staff via influencing definable theoretical determinants of staff behaviour.

## Supplementary information

**Additional file 1.** Questionnaire items with associated behavioural determinants and theories.

## Data Availability

The datasets used and analysed during the current study are available from the corresponding author on reasonable request.

## Abbreviations

BCT: Behaviour Change Technique
COM-B framework: Capability, Opportunity, Motivation, Behaviour framework
ICE: Ideas, Concerns & Expectations
IQR: Interquartile Range
MECC: Making Every Contact Count
NHS: National Health Service
SD: Standard Deviation
TEnT PEGS toolkit: Tailored plans, Environmental change, Thoughts, Practice & record, Emotions, Goals, & Social influences toolkit
