# Supplementary material for: Online behaviour change technique training to support healthcare staff ‘Make Every Contact Count’
Source: BMC Health Serv Res. 2020 May 7;20:390. doi: 10.1186/s12913-020-05264-9 (PMC7206818; doi:10.1186/s12913-020-05264-9)
Supplement: Supplementary file 1 — Additional file 1. Questionnaire items with associated behavioural determinants and theories. [file 12913_2020_5264_MOESM1_ESM.docx]

| **Questionnaire item** | **Response format** | **Behavioural determinant /theoretical construct** | **Theory aligned with item/construct** |
| --- | --- | --- | --- |
| How many conversations with service users to help them make lifestyle changes have you had in the last week? | Provide frequency | Self-reported behaviour | N/A – focus of all behavioural or behaviour change theories |
| I am confident in my ability to have conversations with service users to help them make lifestyle changes | 7-point Likert Scale (strongly disagree – strongly agree) | Self-efficacy  Perceived behavioural control | SCT  TPB |
| People who I work with think that I should be having conversations with service users to help them make lifestyle changes | 7-point Likert Scale (strongly disagree – strongly agree) | Subjective norms | TPB |
| I have sufficient time to have conversation with service users to help them make lifestyle changes | 7-point Likert Scale (strongly disagree – strongly agree) | Opportunity | COM-B |
| For me it is easy to have conversations with service users to help them make lifestyle changes | 7-point Likert Scale (strongly disagree – strongly agree) | Capability  Self-efficacy | COM-B  SCT |
| If I have conversations with service users to help them make lifestyle changes they’re more likely to change their lifestyle than if I don’t | 7-point Likert Scale (strongly disagree – strongly agree) | Outcome expectancies | SCT |
| I have deliberately planned when, where and how to have conversations with service users to help them make lifestyle changes | 7-point Likert Scale (strongly disagree – strongly agree) | Action planning | II |
| When I am working with service users I think about having conversations with them to help make lifestyle changes | 7-point Likert Scale (strongly disagree – strongly agree) | Intention  Habit | TPB  LT |
| It is part of my job to have conversations with service users to help them make lifestyle changes | 7-point Likert Scale (strongly disagree – strongly agree) | Subjective norms  Outcome expectancies | TPB  SCT |
| For every 10 service users you use, with how many would you expect to have a conversation to help them make lifestyle changes? | Select frequency 0-10 | Intention  Motivation | TPB  COM-B |

TPB = Theory of Planned Behvaiour; SCT=Social Cognitive Theory; COM-B = Capability, Opportunity, Motivation – Behvaiour; II = Implementation intentions. LT = Learning Theory.
